# Accumulated lipids rather than the rigid cell walls impede the extraction of genetic materials for effective colony PCRs in Chlorella vulgaris

**DOI:** 10.1186/1475-2859-12-106

**Published:** 2013-11-13

**Authors:** Crystal Jing Ying Tear, Chanyuen Lim, Jinchuan Wu, Hua Zhao

**Affiliations:** 1 Industrial Biotechnology Division, Institute of Chemical & Engineering Sciences, Agency for Science, Technology and Research (A*STAR), 1 Pesek Road, 627833 Jurong Island, Singapore

**Keywords:** Colony PCR protocol, Microalgae, *Chlorella vulgaris*

## Abstract

**Background:**

Failure of colony PCRs in green microalga *Chlorella vulgaris* is typically attributed to the difficulty in disrupting its notoriously rigid cell walls for releasing the genetic materials and therefore the development of an effective colony PCR procedure in *C. vulgaris* presents a challenge.

**Results:**

Here we identified that colony PCR results were significantly affected by the accumulated lipids rather than the rigid cell walls of *C. vulgaris.* The higher lipids accumulated in *C. vulgaris* negatively affects the effective amplification by DNA polymerase. Based on these findings, we established a simple and extremely effective colony PCR procedure in *C. vulgaris*. By simply pipetting/votexing the pellets of *C. vulgaris* in 10 ul of either TE (10 mM Tris/1 mM EDTA) or 0.2% SDS buffer at room temperature, followed by the addition of 10 ul of either hexane or Phenol:Chloroform:Isoamyl Alcohol in the same PCR tube for extraction. The resulting aqueous phase was readily PCR-amplified as genomic DNA templates as demonstrated by successful amplification of the nuclear 18S rRNA and the chloroplast *rbcL* gene. This colony PCR protocol is effective and robust in *C. vulgaris* and also demonstrates its effectiveness in other *Chlorella* species.

**Conclusions:**

The accumulated lipids rather than the rigid cell walls of *C. vulgaris* significantly impede the extraction of genetic materials and subsequently the effective colony PCRs. The finding has the potential to aid the isolation of high-quality total RNAs and mRNAs for transcriptomic studies in addition to the genomic DNA isolation in *Chlorella*.

## Background

Due to their diversity, robustness of growing under brackish water, easiness of cultivation, minimum nutrition requirement and high algal oil content, microalgae are heralded as the 3^rd^ generation feedstock for biofuels production [1-18]. Among those eukaryotic microalgae, the green microalga *Chlorella vulgaris* is the most promising species for industrial applications. In comparison to other microalgae, *C. vulgaris* has relatively high growth rates, photosynthetic efficiencies and capable of accumulating over 50% lipids (dry cell weight) under nitrogen limited conditions [4,5].

Those advantages have led to the great interest in this species for algal oil production and significant efforts have been exercised towards the molecular level understanding and characterization of the lipids pathways and genetic manipulation of *Chlorella* genes for maximizing its oil contents and optimizing the lipid profiles [4,6]. With these goals in consideration, it is essential to develop transformation tools which require a simple procedure for the rapid identification of microbial transformants by simple PCR analysis in routine studies [7]. Due to its rapidness, simplicity and minimal amounts of cells required, colony PCR is an essential and also expeditious technique for such a purpose [5,8]. Besides the identification of microbial transformants, colony PCRs are frequently employed in gene amplification, identification of recombinant integrants and plasmid constructs [9,10], taxonomy screening [11] and diagnostics [12]. In addition, diversity studies of microalgae demands a rapid colony PCR protocol for identification of a collection of algal strains by the 18S rRNA analysis [13].

Few colony PCR procedures have been specifically developed for some microalgae. These methods involve the resuspending of cells using either chelating buffers, 10 nM Ethylenediaminetetraacetic acid (EDTA) [5,14], 5-6% Chelex-100 [5,14] and Tris/EDTA (TE) [5] or in surfactants 0.2% Triton X-100 [14], followed by an essential boiling step to facilitate cell lysis. However, no existing protocols have been successfully applied to *C. vulgaris*. Although Yeast Protein Extraction buffer (Y-PER) was recently employed for colony PCR in *C. vulgaris*[8], it is however more effective in colony PCR of other microalgal strains than *C. vulgaris* and typically requires an additional boiling step for *C. vulgaris*. Furthermore, this additional boiling step leads to the hydrolysis of the cell walls, which results over-release of polysaccharides and subsequently inhibit PCR amplification in *C. vulgaris.* Therefore subsequent dilution of the resulting extract is necessary for effective PCR amplification [8]. Overall, the use of Y-PER in colony PCR of *C. vulgaris* managed to work but required a few additional steps and the colony PCR results are not consistently reproducible.

The rigidity of *C. vulgaris* cell walls is well documented [19,20] and characterized as robust and resilient [21]. In addition, it has been described as a “recalcitrant” and “cumbersome barrier” against oil extraction [22], genetic manipulation plus cell fusion [19,23] and DNA extraction [13], thus requiring the utilization of mechanical homogenization, organic solvents, enzymatic digestion or a combination of these methods [19,20]. Together with the accumulation of lipids in aging *C. vulagris* cultures, its cell walls have been reported to be more resistant to detergents as they age [24]. Therefore, the failure of colony PCR in *C. vulgaris* has been typically attributed to its notoriously rigid cell walls which are considered as the main barrier for effectively releasing of genomic DNA [5,8]. In contrast, here we identified that the accumulated lipids rather than the rigid cell walls affect the effective PCR amplification. Based on it, we established a simple and extremely effective colony PCR procedure for *C. vulgaris* and also demonstrated its effectiveness for colony PCR in other species in the Chlorella genus.

## Materials and methods

### Microalgal strains

Algae culture and the medium used are identical to those ascribed in the UTEX Culture Collection of Algae (http://web.biosci.utexas.edu/utex/) and are as follows. *Chlorella protothecoides* UTEX 256, *Chlorella minutissima* UTEX 2341, *Chlorella kessleri* UTEX 262, and *Chlorella luteoviridis* UTEX 258 were grown in proteose medium, *Chlorella sorokiniana* UTEX 1666 in volvox-dextrose medium, *Chlorella anitrata* UTEX 1798 in modified Bold 3N medium, *Chlorella vulgaris* UTEX 30 in algae culture broth Fluka 17124, *Chlorella desiccata* UTEX 2526 in artificial seawater medium, *Chlorella stigmatophora* UTEX LB993 in Erdschreiber’s medium, *Chlorella saccharophila* UTEX 2911 in Trebouxia Medium, and *Chlorella sphaerica* UTEX LB2485 and *Chlorella* spp. in Bold 3N medium. The detailed compositions of these culture media were described in the UTEX culture collection of Algae (http://web.biosci.utexas.edu/utex/).

### Lysis buffers for cell disruption

Three different lysis buffers, 10 mM Tris (T4661 Sigma Trizma® base) /1 mM Ethylenediaminetetraacetic acid (EDTA) E6758 Sigma (TE buffer), 0.2% Sodium dodecyl sulfate (SDS) (Cat# 102918, MP Biomedicals) and Yeast Protein Extraction buffer (Y-PER) (Product# 78990, Thermo Scientific) are individually examined for their genomic DNA extraction efficiency for colony PCRs. 95% n-hexane (Cat# 650552, Sigma) and Phenol:Chloroform:Isoamyl Alcohol (P:C:I 25:24:1) (P2069, Sigma) are used for lipid extraction.

### Colony PCR procedures

Algal cells collected from a colony or liquid culture were resuspened into 10 μl of the above mentioned buffers in a PCR tube. To facilitate cell disruption, the mixture was pipetted/vortexed for a few seconds, followed by the addition of 10 μl of hexane in the initial testing and later replaced by Phenol:Chloroform:Isoamyl Alcohol (PCI) in the same tube. The lysate was micro-centrifuged at room temperature 25°C and 13.4 × 1000 rpm using Eppendorf Minispin® (Cat# 5452 000.018) and 1-2 μl of the resulting aqueous layer was directly subjected to PCR analysis.

The extraction efficiency of genomic DNA by various buffers was analyzed by PCR amplification of the nuclear 18S rRNA and the chloroplast *rbcL* gene. The universal oligos for amplification of the 18S rRNA include the forward oligo (5′-CCT GGT TGA TCC TGC CAG-3′) and the reverse oligo (5′-A/TTG ATC CTT CT/CG CAG GTT CA-3′) [5]. Amplification of the chloroplast *rbcL* gene was carried out using the oligo pair, the forward primer (5′-GCG GGT GTT AAA GAC TAC CG-3′) and the reverse primer (5′-CCT AAA GTA CCA CCG CCA AA-3′). PCRs were carried out in 20 μl of 1× high fidelity buffer B (New England Biolabs, Ipswich, MA), 2 pmol of each primer, 4 μmol of each dNTP, 1-2 μl of diluted genomic DNA templates extracted previously, and 1 units of Phusion DNA polymerase with high fidelity for 30 cycles using i-Cycler from BioRad (Hercules, CA, USA). Each cycle consisted of 10 s at 98°C, 30 s at 56°C, and 45 s at 72°C, with a final extension step of 5 mins at 72°C. The PCR products were subjected to 0.8% agarose gel electrophoresis for analysis.

### Algal cell culture and total lipid analysis

*C. vulgaris* cells from a colony were picked and inoculated in 100 ml freshwater algae media (Fluka 17124) under sterile conditions and grown in 100 lumens fluorescence lighting using a ratio of 16:8 light to dark cycle and agitation of 100 rpm at 25°C. After 14-days growth, this initial seed culture was re-inoculated into four 200 ml cultures in 1 litre shake flasks and grown in the same conditions above.

Cell density was measured using the difference in absorbance at 735 nm and 680 nm. Cell lysis and triglyceride extraction of *C. vulgaris* were carried out by centrifuging 5 ml of algae culture which was followed by re-suspension of the algae cell pellet in 20 ul of 30% (v/v) Triton-× 100 in ethanol. Post incubation at 100°C for 5 minute, the mixture was microcentrifuged at 13,400 rpm for 2 minutes and the resulting supernatant used for cellular lipid analysis with a commercial serum triglyceride kit (Sigma TR0100), according to the manufacturer’s instructions. Cellular triglyceride was quantified using a glyceryl trioleate (T7140 Sigma) standard curve obtained using the above kit and shown here as relative triglyceride per cell concentration using the above OD values.

## Results

**Figure 1:**
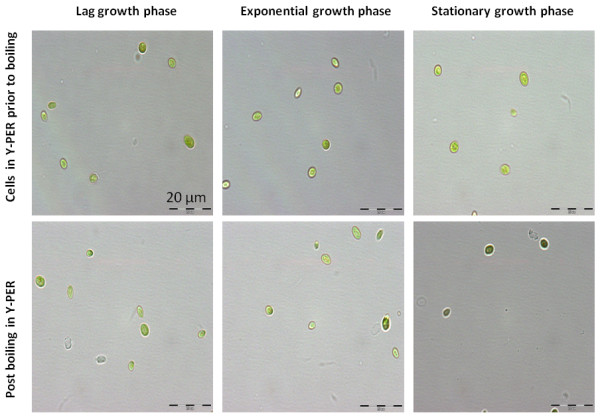
**Microsopoic examination of *C. vulgaris *cells treated with Y-PER reagent.***C. vulgaris* harvested at the different growth stages (upper panel) and post the additional boiling step at 98°C for 5 min (lower panel).

Our recent efforts on the development of transformation protocols in *C. vulgaris* require a procedure for rapid identification of transformants by PCR screening. We previously demonstrated that using of Y-PER with additional boiling step (98°C for 10 mins) led to the effective release of genetic materials from *C. vulgaris*[8] regardless of growth stage. However, we observed that the PCRs frequently failed with the released genomic DNA, especially from the aged cells. Microscopic examination (Figure 1) of the Y-PER treated algal cells harvested from different growth phases further illustrated no morphological differences of the un-deformed rigid cell walls following the elution of DNA. This suggests other factors rather than the rigid cell walls impeding genetic material extraction. Based on those observations, we postulated that factors other than the rigid cell walls impeding genetic material extraction and subsequent PCR amplification. Since *C. vulgaris* is known to accumulate significant lipids in the stationary growth phase, this prompts us to examine if these accumulated lipids impede effective PCR amplification.

**Figure 2:**
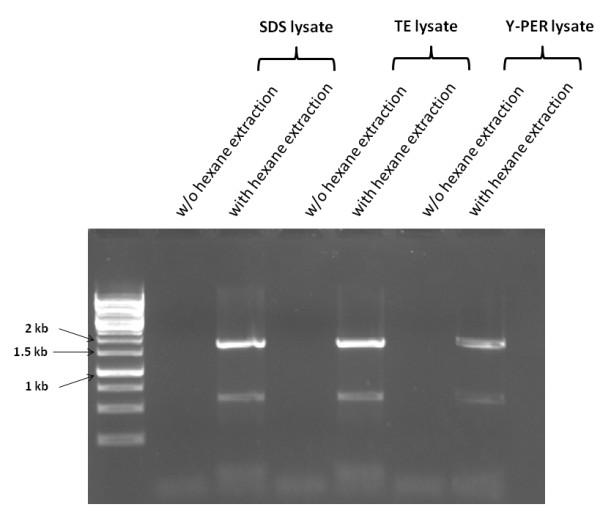
**Colony PCR amplification of the 18S rRNA from the aqueous layer of hexane extracted cell lysate of *C. vulgaris*.**

Using hexane, a solvent known specifically for lipids extraction in microalgae, crude cell lysates of the aged *C. vulgaris* colonies from an agar culture (past 14 days) are disrupted by pipette mixing in TE, 0.2% SDS and Y-PER buffers prior to lipid extraction. Post micro-centrifugation, the resulting aqueous phases were analyzed by PCR amplification. Sequences encoding the 18S rRNA was successfully amplified from the aqueous layer in the respective buffers as indicated in Figure 2, while no PCR products were detected from the crude cell lysates using the various buffers without the additional hexane extraction step. This distinctly demonstrated that the further extraction of the crude cell lysate with hexane was essential. It also suggests that lipid removal by hexane extraction is a necessary step for successful PCRs.

In addition, we observed that amongst those aqueous buffers for initial cell disruption by pipetting, the aqueous phases from the initial 10 mM TE and 0.2% SDS treatment produced better results than Y-PER when used as DNA templates for PCRs. Furthermore, in consideration of the unknown compositions of Y-PER reagent, we employed TE and 0.2% SDS for the cell disruption in all the subsequent experiments. Secondly, due to its high flammability hexane was replaced with PCI for lipid removal. Additionally in contrast to hexane, PCI produces an immiscible layer denser than water, thus allowing simpler and more precise sampling from the aqueous layer. Further removal of residual phenol from the aqueous layer is not required for successful PCR.

**Figure 3:**
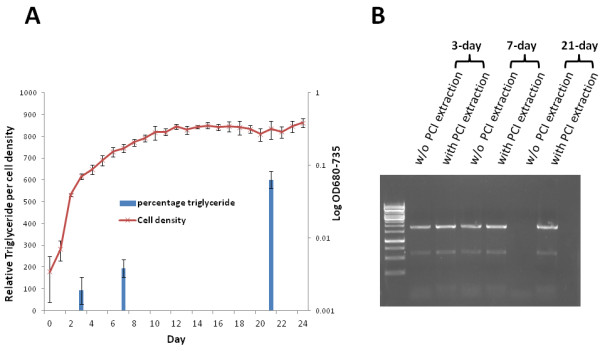
**Total lipids accumulated in various growth stages of *C. vulgaris *(A) and their impact on the effective colony PCR amplifications (B).** PCI denotes Phenol:Chloroform:Isoamyl Alcohol (Cat# P2069, Sigma).

To further evaluate the impact of lipid accumulation in *C. vulgaris* on the efficacy of PCR amplification, we performed PCRs on algal cells extracted under various growth stages. Consistent with the above study, the additional step with PCI extraction led to successful PCR amplifications of the 18S rRNA sequences regardless of the growth stages (Figure 3). We also noticed that further PCI extraction is not required for successful PCR amplification of the 18S rRNA sequences from crude cell lysate disrupted in TE harvested in both the lag phase and the exponential growth phase, which corresponded to the trace lipid accumulation as indicated in Figure 3. However, the removal of total lipids by the additional PCI extraction was essential for the cell lysates from the stationary growth phase which accumulated relatively higher lipids (Figure 3). These results collectively demonstrate that the removal of accumulated lipids is essential for successful colony PCRs.

**Figure 4:**
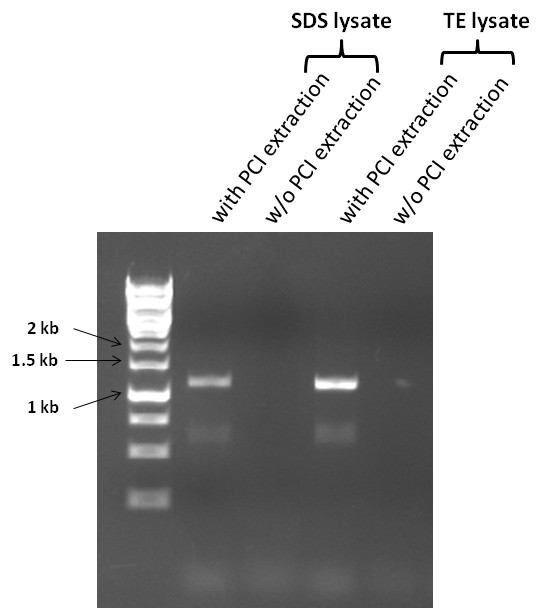
**PCR amplification of the chloroplast *rbcL *gene from the aqueous layer of phenol-chloroform extracted cell lysate of *C. vulgaris*.** PCI denotes Phenol:Chloroform:Isoamyl Alcohol (Cat# P2069, Sigma).

In addition to the nuclear 18S rRNA analysis, we next examined the effectiveness of PCR amplifying the chloroplast *rbcL* gene in *C. vulgaris* from the aqueous layer of the extracts. As indicated in Figure 4, *rbcL* gene was successfully amplified from the aqueous layer of the TE crude lysate. This suggests the overall effectiveness of the initial cell disruption by agitation in TE buffer and the subsequent lipid removal by PCI extraction on releasing both the nuclear and chloroplast DNAs.

**Figure 5:**
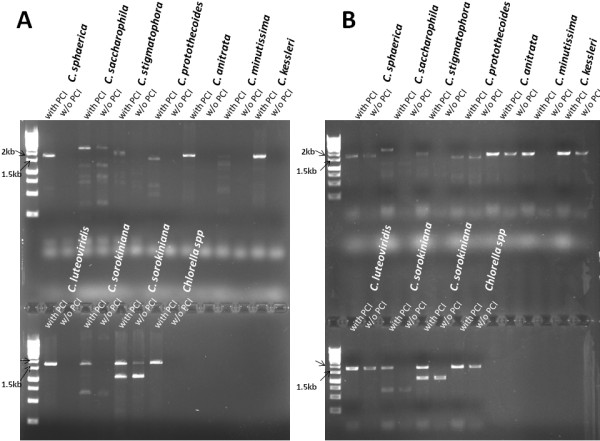
**Colony PCR analysis of the 18S rRNA from other species of *Chlorella*.** Cells were disrupted in either 10 mM TE **(A)** or 0.2% SDS **(B)** and followed by PCI extraction for lipid removal. PCI denotes Phenol:Chloroform:Isoamyl Alcohol (Cat# P2069, Sigma).

We next asked if this procedure is effective in other species of *Chlorella*. By following the above protocol, strains from various *Chlorella* species were agitated in either TE or 0.2% SDS and followed by lipids removal using PCI extraction, the extracted genomic DNAs in the aqueous layers were subjected to the 18S rRNA PCR amplification. In contrast with those crude cell lysates without further PCI extraction, the 18S rRNAs sequences were successfully amplified from all the test strains as depicted in Figure 5. This result further demonstrated the effectiveness of the additional PCI treatment of the crude cell lysates on the isolation of PCR-quality genomic DNA from a wide range of *Chlorella* species. Based on Figure 5, we also observed that the initial step of disruption of algal cells in either TE or 0.2% SDS were comparably inadequate for the releasing genomic DNAs by agitation and this further suggests that the subsequent phenol-chloroform extraction results the isolation of PCR-quality genomic DNAs.

## Discussion

As the most promising eukaryotic green microalga with the potential for industrial application for algal oil production, intensive research efforts have been attempted on *C. vulgaris* amongst other oleaginous algal species and therefore it is essential to develop a simple PCR protocol for rapid identification of recombinants with desired phenotypes. In this work, we identified the accumulated lipids (removed via solvent extraction) rather than the rigid cell walls which are often considered in previous researches [13,19,23] as the major barriers impeding DNA extraction. Based on this finding, we established a robust protocol for simple, effective and robust colony PCR in *C. vulgaris*.

Previous protocols [5,14] reported the use of the chelating buffers, 10 mM EDTA, 5-6% Chelex-100 and TE buffer, together with the use of the surfactants, 0.2% Triton X-100. The ionic detergent SDS and Triton X-100 are used to cause disruption of the cell membrane, resulting in the lysis and release of cellular components [25,26], while the use of chelating agents prevent cellular nuclease degradation of nucleic acids during cell disruption [25,27]. These extraction buffers do not have the properties for oil removal. The organic solvent Phenol:Chloroform [27] were used mainly to separate cellular and nucleoproteins from DNA. By contrast, hexane [28] and chloroform–methanol [22] have been previously used for lipid extraction from microalgae. In the manuscript presented here, hexane in conjunction with TE buffer was initially used for lipid extraction/separation from *C. vulgaris* without prior mechanical disruption and DNA was obtained from the lower aqueous TE buffer phase of the immiscible mixture post centrifugation. This was later substituted with Phenol:Chloroform:Isoamyl Alcohol due to it being denser than the aqueous extraction buffers, allowing a cleaner and more rapid removal of the aqueous phrase containing DNA. An additional comparison of rapid DNA extraction without boiling comparing TE buffer, 0.2% SDS, Y-PER [8] and PCI was carried out on aged *C. vulgaris* cultures. Of these, PCR amplification was successful only with the use of PCI extraction as described in this work (Additional file 1: Figure S1).

In addition, Modifications of the DNA extraction using Chelex-100 [14] involve additional mechanical homogenization for higher plants [29] or high temperature incubation in ethanol following homogenization for *Chlamydomonas reinhardtii* and *Arabidopsis thaliana*[30]. The protocol presented here dispenses with these procedures, involves fewer steps and equipment. Compared with the use of Chelex-100, a chelating resin in combination with incubation in ethanol, the method here clearly segregates the genetic material from cellular lipids and non polar cell components. Additionally, incubation at high temperatures (100°C) for at least 10 minutes was necessary for the release of chloroplastic genetic material for the above method [5]. In the protocol presented here, DNA from chloroplast is obtained in a single step and without the high temperature or boiling step. DNA degradation can thus be reduced [31] and the procedure may be readily adaptable for the extraction of other cellular components. The study [5] described the use of Chelex-100 for extracting PCR-quality genomic DNAs from *Chlorella* species. Its effectiveness was demonstrated in the *Chlorella* species, *C. sorokiniana*, *C. protothecoides*, *C. zofingiensis* and *C. kessleri*. However, DNA extraction of *C. vulgaris* was not carried out nor described in the references [5,14].

To the best of our knowledge the only example of rapid DNA extraction in *C. vulgaris*[8] requires boiling incubation, subsequent centrifugation of the cell pellet in the surfactant, Y-PER and dilution of the supernatant in water. No other procedure involving the use of Chelex-100 on DNA extraction of *C. vulgaris* has been reported [14]. Fundamental differences exist in the cell wall composition [19,20], rigidity and cellular components of *Chlamydomonas reinhardtii, a* model organism for microalgae study and *C. vulgaris* with its lipid accumulating potential, thus requiring the use of different regime for DNA extraction.

In summary, we have established here, a simple and extremely efficient method for routine colony PCR analysis in *C. vulgaris* that can be extended to other species in Chlorella genus. Additionally, the finding that the accumulated lipids rather than the rigid cell walls negatively affecting effective DNA manipulation has the potential to aid the isolation of high quality and large quantity of total RNAs and mRNAs for transcriptomic studies in addition to the genomic DNA isolation in *Chlorella*.

## Competing interests

The authors declare that they have no competing interests.

## Authors’ contributions

CJYT and CL performed laboratory work. JW analyzed the data. HZ conceived the experiment and analyzed the data. CL and HZ drafted the manuscript. All authors read and approved the final manuscript.

## Supplementary Material

Additional file 1: Figure S1 A comparison of rapid DNA extraction from *C. vulgaris* harvested in stationary growth phase without boiling using individual buffers for PCR amplification. The buffers, 10 mM Tris/1 mM EDTA (TE), 0.2% Sodium dodecyl sulfate (SDS), Yeast Protein Extraction buffer (Y-PER) (Cat# 78990, Thermo Scientific), Chelex-100, and TE treated followed by PCI extraction were examined for their genomic DNA extraction efficiency for colony PCRs. Click here for file

## References

[B1] Mata TM Martins AA Caetano NS Microalgae for biodiesel production and other applications: a review Renew Sustain Energy Rev 2010 14 217 232 10.1016/j.rser.2009.07.020

[B2] Chisti Y Biodiesel from microalgae Biotechnol Adv 2007 25 294 306 10.1016/j.biotechadv.2007.02.001 17350212

[B3] Yu WL Ansari W Schoepp NG Hannon MJ Mayfield SP Burkart MD Modifications of the metabolic pathways of lipid and triacylglycerol production in microalgae Microb Cell Fact 2011 10 91 10.1186/1475-2859-10-91 22047615 PMC3234195

[B4] Liang Y Sarkany N Cui Y Biomass and lipid productivities of Chlorella vulgaris under autotrophic, heterotrophic and mixotrophic growth conditions Biotechnol Lett 2009 31 1043 1049 10.1007/s10529-009-9975-7 19322523

[B5] Wan M Rosenberg JN Faruq J Betenbaugh MJ Xia J An improved colony PCR procedure for genetic screening of Chlorella and related microalgae Biotechnol Lett 2011 33 1615 1619 10.1007/s10529-011-0596-6 21431847

[B6] Stephenson AL Dennis JS Howe CJ Scott SA Smith AG Influence of nitrogen-limitation regime on the production by Chlorella vulgaris of lipids for biodiesel feedstocks Biogeosciences 2010 1 47 58

[B7] Radakovits R Jinkerson RE Darzins A Posewitz MC Genetic engineering of algae for enhanced biofuel production Eukaryot Cell 2010 9 486 501 10.1128/EC.00364-09 20139239 PMC2863401

[B8] Packeiser H Lim CY Balagurunathan B Wu JC Zhao H An extremely simple and effective colony PCR procedure for bacteria, yeasts, and microalgae Appl Biochem Biotechnol 2013 169 695 700 10.1007/s12010-012-0043-8 23271627

[B9] Luhe AL Gerken H Tan L Wu JC Zhao H Alcohol tolerance of Escherichia coli acrR and marR regulatory mutants J Mol Cat B: Enzymatic 2012 76 89 93

[B10] Cairney J Xu N Pullman GS Ciavatta VT Johns B Natural and somatic embryo development in loblolly pine Appl Biochem Biotechnol 1999 77 5 17 10.1385/ABAB:77:1-3:5

[B11] Niemi RM Heiskanen I Wallenius K Lindstrom K Extraction and purification of DNA in rhizosphere soil samples for PCR-DGGE analysis of bacterial consortia J Microbiol Methods 2001 45 155 165 10.1016/S0167-7012(01)00253-6 11348673

[B12] Müller FM Werner KE Kasai M Francesconi A Chanock SJ Walsh TJ Rapid extraction of genomic DNA from medically important yeasts and filamentous fungi by high speed cell disruption J Clin Microbiol 1998 36 1625 1629 9620390 10.1128/jcm.36.6.1625-1629.1998 PMC104890

[B13] Fawley KP Fawley MW A simple and rapid technique for the isolation of DNA from microalgae J Phycol 2004 40 223 225 10.1111/j.0022-3646.2004.03-081.x

[B14] Cao M Fu Y Guo Y Pan JM Chlamydomonas (Chlorophyceae) colony PCR Protoplasma 2009 235 107 110 10.1007/s00709-009-0036-9 19242652

[B15] Schultz DJ Craig R Cox-Foster DL Mumma RO Medford JI RNA isolation from recalcitrant plant tissue Plant Mol Bio Rep 1994 12 310 316 10.1007/BF02669273

[B16] Snirc A Silberfeld T Bonnet J Tillier A Tuffet S Sun JS Optimization of DNA extraction from brown algae (Phaeophyceae) based on a commercial kit J Phycol 2010 46 616 621 10.1111/j.1529-8817.2010.00817.x

[B17] Falcao VDR Tonon AP Oliveira MC Colepicolo P RNA Isolation method for polysaccharide rich algae: agar producing Gracilaria tenuistipitata(Rhodophyta) J Appl Phycol 2008 20 9 12 10.1007/s10811-007-9174-7

[B18] Lim DKY Garg S Timmins M Zhang ESB Thomas-Hall SR Schuhmann H Li Y Schenk PM Isolation and evaluation of oil-producing microalgae from subtropical coastal and brackish waters PLoS ONE 2012 7 e40751 10.1371/journal.pone.0040751 22792403 PMC3394722

[B19] Coll JM Review-methodolgies for transferring DNA into eukaryotic microalgae Spanish J Agr Res 2006 4 316 330

[B20] Gerken HG Donohoe B Knoshaug EP Enzymatic cell wall degradation of Chlorella vulgaris and other microalgae for biofuels production Planta 2013 237 239 53 10.1007/s00425-012-1765-0 23011569

[B21] Atkinson JAW Gunning BES John PCL Sporopollenin in the cell wall of Chlorella and other algae: ultrastructure, chemistry, and incorporation of 14C-acetate, studied in synchronous cultures Planta 1972 107 1 32 10.1007/BF00398011 24477346

[B22] Zheng H Yin J Gao Z Huang H Ji X Dou C Disruption of Chlorella vulgaris Cells for the release of biodiesel-producing lipids: a Comparison of grinding, ultrasonication, bead milling, enzymatic lysis, and microwaves Appl Biochem Biotechnol 2011 164 1215 1224 10.1007/s12010-011-9207-1 21347653

[B23] Honjoh KI Suga K Shinohara F Maruyama I Miyamoto T Hatano S Iio M Preparation of protoplasts from Chlorella vulgaris K-73122 and cell wall regeneration of protoplasts from C. vulgaris K-73122 and C-27 J Fac Agric Kyushu Univ 2003 47 257 266

[B24] Corre G Templier J Largeau C Rousseau B Berkaloff C Influence of cell wall composition on the resistance of two Chlorella species (chlorophyta) to detergents J Phyco 1996 32 584 590 10.1111/j.0022-3646.1996.00584.x

[B25] Marmur J A procedure for the isolation of deoxyribonucleic acid from micro-organisms J Mol Biol 1961 3 208 218 10.1016/S0022-2836(61)80047-8

[B26] Birnboim HC Doly J A rapid alkaline extraction procedure for screening recombinant plasmid DNA Nucleic Acids Res 1979 7 6 10.1093/nar/7.6.1513 PMC342324 388356

[B27] Dale JW Greenaway PJ Preparation of chromosomal DNA from E. coli Methods Mol Biol 1985 2 197 200 21374192 10.1385/0-89603-064-4:197

[B28] Halim R Gladman B Danquah MK Webley PA Oil extraction from microalgae for biodiesel production Bioresour Technol 2011 102 178 85 10.1016/j.biortech.2010.06.136 20655746

[B29] HwangBo K Son SH Lee JS Min SR Ko SM Liu JR Choi DS Jeong WJ Rapid and simple method for DNA extraction from plant and algal species suitable for PCR amplification using a chelating resin Chelex 100 Plant Biotechnol Rep 2010 4 49 52 10.1007/s11816-009-0117-4

[B30] Berthold DA Best BA Malkin R A rapid DNA preparation for PCR from Chlamydomonas reinhardtii and Arabidopsis thaliana Plant Mol Biol Report 1993 11 338 344 10.1007/BF02905336

[B31] Walsh PS Metzger DA Higuchi R Chelex 100 as a medium for simple extraction of DNA for PCR-based typing from forensic material Biotechniques 1991 10 506 513 1867860

