# Supplementary material for: Accumulated lipids rather than the rigid cell walls impede the extraction of genetic materials for effective colony PCRs in Chlorella vulgaris
Source: Microb Cell Fact. 2013 Nov 13;12:106. doi: 10.1186/1475-2859-12-106 (PMC3835426; doi:10.1186/1475-2859-12-106)
Supplement: Additional file 1: Figure S1 — A comparison of rapid DNA extraction from C. vulgaris harvested in stationary growth phase without boiling using individual buffers for PCR amplification. The buffers, 10 mM Tris/1 mM EDTA (TE), 0.2% Sodium dodecyl sulfate (SDS), Yeast Protein Extraction buffer (Y-PER) (Cat# 78990, Thermo Scientific), Chelex-100, and TE treated followed by PCI extraction were examined for their genomic DNA extraction efficiency for colony PCRs. [file 1475-2859-12-106-S1.docx]

**Supplementary data**


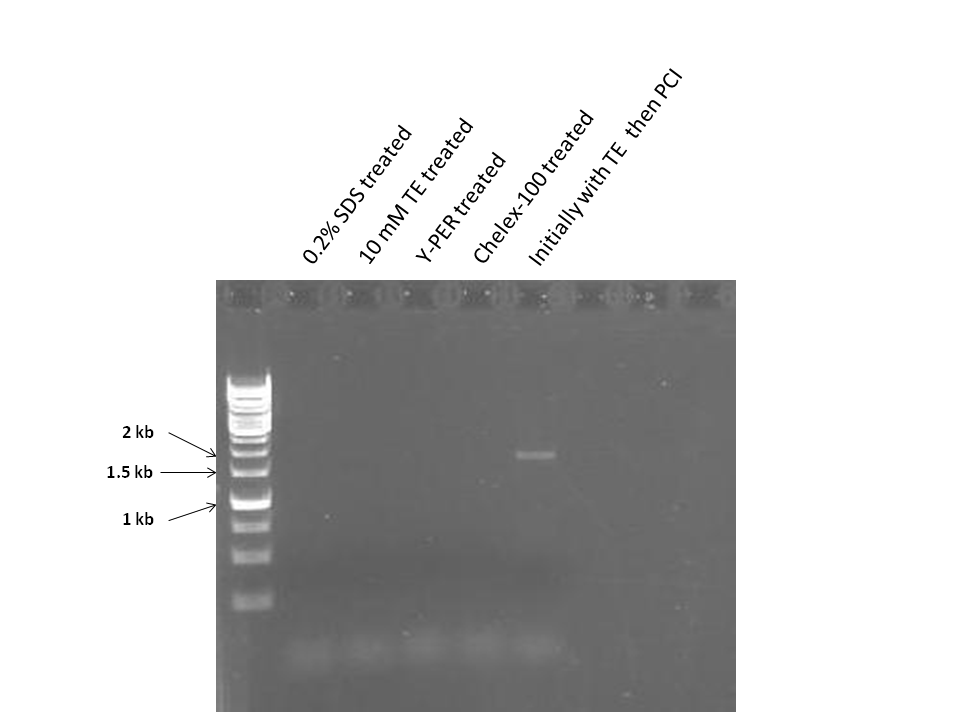


**Fig. S1.** A comparison of rapid DNA extraction from *C. vulgaris* harvested in stationary growth phase without boiling using individual buffers for PCR amplification. The buffers, 10 mM Tris /1 mM EDTA (TE), 0.2% Sodium dodecyl sulfate (SDS), Yeast Protein Extraction buffer (Y-PER) (Cat# 78990, Thermo Scientific), Chelex-100, and TE treated followed by PCI extraction were examined for their genomic DNA extraction efficiency for colony PCRs.
